# Prevalence of depression and analysis of its association with anxiety in military police officers: a cross-sectional study

**DOI:** 10.1590/0034-7167-2024-0312

**Published:** 2025-12-08

**Authors:** Renato Canevari Dutra da Silva, Carolina Veras Mendes, Maria Neyrian de Fátima Fernandes, Gilson Gonçalves Silva, Lidiane Bernardes Faria Vilela, Elton Brás Camargo

**Affiliations:** I Universidade de Rio Verde. Rio Verde, Goiás, Brazil; II Universidade Federal do Maranhão. Imperatriz, Maranhão, Brazil

**Keywords:** Police, Mental Health, Depression, Anxiety, Mental Disorders., Policía, Salud Mental, Depresión, Ansiedad, Trastornos Mentales.

## Abstract

**Objective:**

to identify the prevalence of depression and its association with anxiety levels among military police officers.

**Methods:**

a cross-sectional study, conducted with active military police officers, using the Beck Depression Inventory and the Beck Anxiety Inventory, both validated for the Brazilian context. Multivariate logistic regression analyses verified associations.

**Results:**

the sample consisted of 672 military police officers, with a prevalence of depression of 34.5%. Police officers with depression presented significantly higher levels of anxiety. Anxiety was a significant predictor of depression, with each one-point increase in anxiety score being associated with a 1.20-fold increase in the odds of depression (adjusted Odds Ratio = 1.20; 95% Confidence Interval 1.16-1.24; p < 0.001).

**Conclusions:**

the high prevalence of depression and its association with anxiety highlight the need to implement prevention and mental health promotion actions among military police officers.

## RESUMEN

**Objetivos:**

identificar la prevalencia de depresión y su asociación con los niveles de ansiedad en policías militares.

**Métodos:**

estudio transversal realizado con policías militares en activo, utilizando el Inventario de Depresión de Beck y el Inventario de Ansiedad de Beck, ambos validados para el contexto brasileño. Los análisis de regresión logística multivariante verificaron las asociaciones.

**Resultados:**

la muestra estuvo compuesta por 672 policías militares, con una prevalencia de depresión del 34,5%. Los policías con depresión presentaron niveles significativamente más altos de ansiedad. La ansiedad fue un predictor significativo de depresión, ya que cada aumento de un punto en la puntuación de ansiedad se asoció con un aumento de 1,20 veces en la probabilidad de depresión (Odds Ratio ajustada = 1,20; Intervalo de Confianza del 95%: 1,16-1,24; p < 0,001).

**Conclusiones:**

la alta prevalencia de depresión y su asociación con la ansiedad resaltan la necesidad de implementar acciones de prevención y promoción de la salud mental entre los agentes de policía militar.

## INTRODUCTION

The military police profession can impose a significant psychological burden. Every police call is imbued with the prospect of encountering emotionally challenging situations, exemplified by frequent traumatic calls for service, including incidents such as child abuse, domestic violence, car accidents, and homicides^(1)^. Therefore, it is imperative to recognize that a stressful occupational environment has the potential to precipitate several physical and psychological health complications.

Scientific evidence^(1,2)^ has shown that police officers experience some form of mental health problem while on the job, but even those who report frequent stress and poor mental health are unlikely to request time off work because of the stigma attached to mental illness in the force. Moreover, police officers often experience symptoms of depression and anxiety^(3)^. The presence of high levels of anxiety and depressive symptoms can result in serious consequences, such as suicide. Data indicate that suicide among police officers is a serious public health problem, with 85 cases recorded in 2022 among active military police officers^(4)^.

Mental overload also exerts an influence on the body and can manifest itself through physical symptoms, such as an increase in occupational injuries and the number of stress-related diagnoses, such as high blood pressure or gastrointestinal ulcers. Police stressors and their mental and physical implications harm the police organization in the long term, resulting in reduced performance, increased absenteeism, and increased police aggression^(5)^.^.^

There is still a lack of research on military police officers’ psychological problems in Brazil, and there is a need to conduct research that assesses the factors that influence police officers’ mental health^(6)^. Therefore, conducting research to learn more about this population’s mental health profile is essential. A study of this nature contributes to the limited body of existing literature investigating the prevalence of depression and anxiety in military police officers.

## OBJECTIVES

To identify the prevalence of depression and its association with anxiety among military police officers.

## METHODS

### Ethical aspects

This research was approved by the Research Ethics Committee of the proposing institution, and followed the ethical precepts established by Resolutions 466/2012 and 588/2018. Research participants provided their consent by signing the Informed Consent Form.

### Study design, period and place

This is a cross-sectional, inferential and quantitative study, carried out with active military police officers working in the state of Goiás. The state of Goiás, located in the Central-West region of Brazil, has a population of 7,056,495 inhabitants, being the 11^th^ most populous state in the country and having a contingent of 10,987 military police officers. The data collection period took place from December 2021 to January 2022.

The convenience sample was recruited from the 18 Regional Commands (In Portuguese, *Comandos Regionais de Polícia Militar* - CRPM) of Goiás, which cover all state microregions. The survey was conducted online based on the STrengthening the Reporting of OBservational studies in Epidemiology and CHEcklist for Reporting Results of Internet E-Surveys.

### Population or sample; inclusion and exclusion criteria

The eligibility criteria for the study were to be military police officers of Goiás who were active and held positions in one of the 18 CRPM. The exclusion criterion was defined as police officers who reported being currently undergoing treatment for a mental disorder. Since this was a convenience sample, the sample power was assessed after data collection. To calculate the sample power, the analyses of logistic regression statistics were taken into account, assuming that anxiety has a moderate influence on depression (Odds Ratio = 1.2) and a proportion of depression of around 20%. The sample obtained in the present study was 672 police officers in a population of 10,987 police officers in the state of Goiás, and when considering these variables, the sample power result was 97%, which means that the study has a high probability of detecting a significant effect, if it exists, with a 95% confidence level.

### Study protocol

Data collection was carried out through a digital platform. The invitation to participate in the survey was sent to all commanders responsible for the 18 CRPMs in Goiás. Commanders, in turn, were responsible for forwarding information regarding the survey procedures and the electronic address for filling out the forms to the police officers under their jurisdiction.

The data collection instruments were structured on a digital platform (Google Forms^®^) with the assessments organized into three chunks: sociodemographic, work, clinical and lifestyle characteristics; Beck Depression Inventory II; Beck Anxiety Inventory.

As for sociodemographic, work, clinical and lifestyle characteristics, the instrument was developed by the research authors with sociodemographic questions about age, sex, and marital status. Work variables were assessed by identifying the rank, function held in the corporation, service time and income. Police officers’ lifestyle was assessed by physical activity (activity practiced for at least 30 minutes), alcohol and tobacco use. Regarding clinical aspects, the presence of chronic disease and medication daily use were assessed.

Beck Depression Inventory II (BDI-II): depression was assessed using the BDI-II, an instrument validated for the Brazilian context and widely used to assess the severity of depression in clinical and non-clinical samples^(7)^. The instrument consists of 21 questions that assess symptoms such as sadness, guilt, hopelessness, changes in sleep and appetite, among others, reflecting the somatic, cognitive and affective components of depression. The instrument’s score ranges from 0 to 63, depending on the intensity of the symptom experienced in the last week, with higher scores indicating greater severity of depression. As indicated by Gomes-Oliveira *et al.* ^(7)^, military police officers were categorized into two groups based on the BDI-II: absence of depression, with scores equal to or less than 10, and presence of depression, with scores equal to or greater than 11. The reliability results of the instrument in the present sample demonstrated a Cronbach’s alpha coefficient of 0.89 and a McDonald’s omega coefficient of 0.89.

Beck Anxiety Inventory (BAI): anxiety was assessed by the BAI, which is an instrument consisting of 21 items that describe symptoms of anxiety, such as fear, tremors, sweating, nervousness, feelings of panic, among others. The BAI was validated for Brazil, and each instrument item is scored from 0 to 3, where 0 indicates absence of symptoms and 3 indicates severe symptoms^(8)^. The score ranges from 0 to 63, with higher scores reflecting higher levels of anxiety. Due to the latent nature of anxiety and its variation at different levels, it was assessed as a continuous variable. The BAI reliability tests, in the present study, presented Cronbach’s alpha coefficients of 0.92 and McDonald’s omega coefficients of 0.93.

### Analysis of results, and statistics

The database was constructed using a double entry strategy in Excel^®^ and subsequently exported to R Studio version 2024.04.1. The analyses performed included descriptive statistics by presenting absolute (n) and relative (%) frequencies, in addition to the mean (M) and standard deviation (±SD) of continuous variables. To assess the differences between the means of anxiety levels, the Mann-Whitney and Kruskal-Wallis tests were performed, since they did not present a normal distribution. The chi-square test was performed to analyze the associations between depression and independent variables. Significant differences and associations were considered at a significance level of 5% (p < 0.05).

In order to discern the unique impact of anxiety on depression, a multivariate analysis was performed, controlling for variables associated with depression in bivariate analyses. The magnitude of the relationship between anxiety and depression was assessed by multivariate logistic regression, using the adjusted Odds Ratio (aOR) and a 95% Confidence Interval (95%CI), in which p-values less than 0.05 were considered statistically significant. The assumptions of multivariate logistic regression were assessed using the variance inflation factor (VIF) and tolerance metrics. The VIF results ranged from 1.01 to 1.51, and those of tolerance ranged from 0.65 to 0.98, thus meeting the assumptions of multivariate logistic regression.

## RESULTS

The sample of this study consisted of 672 active military police officers from the state of Goiás, Brazil. Most participants were male (90.62%), predominantly between 30 and 39 years of age (47.32%), married (77.53%), and working mainly in operational roles (76.78%), with more than six years of service (76%). The majority of participants earned between R$5,000.00 and R$10,000.00 (65.32%). Concerning physical activity, 21.57% reported not practicing any level of physical activity. Current tobacco use was reported by 11.75%, and alcohol use by 88.24% of the sample assessed. The majority did not have chronic diseases (68.45%) or need daily medication (71.87%). Concerning depression, 232 (34.5%; 95%CI 31-37.9) were categorized as having depression, and 440 (65.5%; 95%CI 62.1-69) did not present significant symptoms of depression. The depression and anxiety scores had averages of 9.47 (±7.94) and 10.37 (±9.55), respectively (Table 1).

**Table 1:** Sociodemographic, work, clinical and lifestyle characteristics of military police officers, Goiás, Brazil, 2021-2022

Variables	n	%
Sex		
Female	63	9.37
Male	609	90.62
Age		
Up to 29 years	65	9.67
30 to 39 years	318	47.32
40 years or older	289	43
Marital status		
Married	521	77.53
Single, divorced or widowed	151	22.47
Police rank		
Enlisted men (soldier to midshipman)	561	83.5
Officers (lieutenant to colonel)	111	16.5
Current police function		
Administrative	156	23.21
Operational	516	76.78
Police service time		
Up to 5 years	161	24
6 years or more	511	76
Income		
Up to R$5,000.00	112	16.66
R$5,000.00 to R$10,000.00	439	65.32
R$10,000.00 to R$15,000.00	66	9.82
R$15,000.00 or more	55	8.18
Physical activity		
Three or more times a week	283	42.11
Up to twice a week	244	36.31
Does not practice	145	21.57
Tobacco use		
No	593	88.24
Yes	79	11.75
Alcoholic beverage use		
No	268	39.88
Yes	404	60.11
Chronic disease		
Does not have	460	68.45
Hypertension	61	9.07
Diabetes mellitus	11	1.63
Others	140	20.83
Medication daily use		
No	483	71.87
Yes	189	28.12
Depression		
Absent	440	65.5
Present	232	34.5
-	**Mean**	**Standard deviation**
BDI score	9,47	7.94
BAI score	10,37	9.55

*BDI - Beck Depression Inventory; BAI - Beck Anxiety Inventory.*

The analyses of the differences in mean anxiety scores, assessed by the BAI, and the associations with depression, assessed by the BDI, are presented in Table 2. Regarding anxiety, women presented a significantly higher mean (13.44; ±9.11) than men (10.04; ±9.55) (p < 0.001). Military police officers aged 40 years or older had significantly higher scores when compared to younger ones (p = 0.04). There was a difference in the mean anxiety scores in relation to the frequency of physical activity (p < 0.001), with those who did not practice physical activity having higher scores (14.40; ± 20.93) when compared to those who practiced it. Police officers who reported using tobacco had a significantly higher mean (22.67; ± 9.02) compared to non-smokers (10.19; ± 9.61) (p = 0.04). The presence of chronic disease and medication daily use was associated with significantly higher levels of anxiety (p < 0.001). In relation to depression, an association was observed with sex, physical activity, chronic disease and medication daily use (p < 0.05) (Table 2).

**Table 2:** Differences in mean anxiety scores and associations with depression according to sociodemographic, work, clinical and lifestyle characteristics of military police officers, Goiás, Brazil, 2021-2022

Variables	Anxiety		Depression		*p* value^*^
	Mean ()	*p* value^*^	Absent n (%)	Present n (%)	
Sex		<0.001			0.04
Female	13.44 (9.11)		34 (7.7)	29 (12.50)	
Male	10.04 (9.55)		406 (92.27)	203 (87.05)	
Age		0.04			0.58
Up to 29 years	8.84 (7.02)		46 (10.45)	19 (8.19)	
30 to 39 years	9.76 (9.09)		204 (46.36)	114 (49.13)	
40 years or older	11.37 (10.42)		190 (43.18)	99 (42.67)	
Marital status		0.52			0.57
Married	10.33 (9.26)		344 (78.18)	177 (76.29)	
Single, divorced or widowed	10.16 (10.52)		96 (21.81)	55 (23.70)	
Police rank		0.09			0.06
Enlisted men (soldier to midshipman)	10.57 (9.59)		359 (81.59)	202 (87.06)	
Officers (lieutenant to colonel)	9.29 (9.30)		81 (18.40)	30 (12.93)	
Current police function		0.30			0.54
Administrative	10.92 (9.37)		99 (22.50)	57 (24.56)	
Operational	10.19 (9.61)		341 (77.50)	175 (75.43)	
Police service time		0.76			0.93
Up to 5 years	9.60 (8.18)		105 (23.86)	56 (24.13)	
6 years or more	10.60 (9.94)		335 (76.13)	176 (75.86)	
Income		0.07			0.13
Up to R$5,000.00	11.83 (10.49)		65 (14.77)	47 (20.25)	
R$5,000.00 to R$10,000.00	10.30 (9.17)		287 (65.22)	152 (65.51)	
R$10,000.00 to R$15,000.00	9.72 (10.31)		48 (10.90)	18 (7.75)	
R$15,000.00 or more	8.63 (9.43)		40 (9.09)	15 (6.46)	
Physical activity		<0.001			<0.001
Three or more times a week	8.43 (8.23)		203 (46.13)	80 (34.48)	
Up to twice a week	10.20 (9.40)		163 (37.04)	81 (34.91)	
Does not practice	14.40 (10.93)		74 (16.81)	71 (30.60)	
Tobacco use		0.04			0.14
No	10.19 (9.61)		394 (89.54)	199 (85.77)	
Yes	22.67 (9.02)		46 (10.55)	33 (14.22)	
Alcoholic beverage use		0.47			0.05
No	10.13 (9.52)		187 (42.50)	81 (34.91)	
Yes	10.51 (9.58)		253 (57.50)	151 (65.08)	
Chronic disease		<0.001			<0.001
Does not have	8.36 (8.14)		340 (77.27)	120 (51.72)	
Hypertension	12.59 (10.37)		38 (8.63)	23 (9.91)	
Diabetes mellitus	15.72 (12.24)		6 (1.63)	5 (2.15)	
Others	15.56 (10.93)		56 (12.72)	84 (36.20)	
Medication daily use		<0.001			<0.001
No	8.63 (8.39)		347 (78.86)	136 (58.62)	
Yes	14.79 (10.85)		93 (21.13)	96 (41.37)	

* p value for Mann-Whitney U or Kruskal-Wallis test;

** p value for chi-square test.

**Figure 1:**
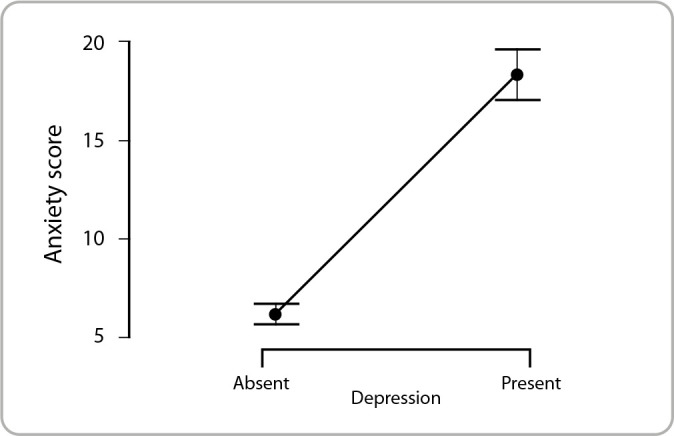
Relationship between depression and anxiety scores in military police officers, Goiás, Brazil, 2021-2022

*Note: circles indicate mean scores, and upper and lower bars indicate 95% Confidence Intervals for each group of officers.*

Figure 1 illustrates the difference between the mean anxiety scores among military police officers according to the absence or presence of depression. Police officers with depression had a significantly higher mean anxiety (18.33; ±10.28) when compared to police officers without depression (6.16; ±5.71) (p < 0.001).

Independent variables significantly associated with depression were entered into a multivariate model in order to investigate their relationships. The presence of other chronic diseases was shown to be a significant predictor of depression. The results of the multivariate logistic regression demonstrated that, even after the inclusion of possible confounding factors, anxiety continues to be a significant predictor of depression. Each one-unit increase in the anxiety score is associated with a 1.20-fold increase in the odds of depression among military police officers (aOR = 1.20; 95%CI 1.16-1.24; p < 0.001) (Table 3).

**Table 3:** Multivariate logistic regression analysis of the association between depression and anxiety controlling for sociodemographic, clinical and lifestyle characteristics, Goiás, Brazil, 2022

Variables	ORa (IC95%)	Valor de *p*
Sex		
Female	1.12 (0.58 - 2.17)	0.72
Physical activity		
Up to twice a week	0.89 (0.55 - 1.42)	0.63
Does not practice	1.11 (0.64 - 1.92)	0.70
Chronic disease		
Hypertension	0.81 (0.35 - 1.88)	0.63
Diabetes mellitus	0.88 (0.16 - 4.65)	0.88
Others	2.13 (1.23 - 3.68)	0.007
Medication daily use		
Yes	1.05 (0.59 - 1.85)	0.86
Anxiety		
BAI score	1.20 (1.16 - 1.24)	<0.001

*aOR - adjusted Odds Ratio; 95%CI - 95% Confidence Interval.*

## DISCUSSION

The results of the study demonstrated a significant prevalence of depression among military police officers. Police officers with depression had significantly higher levels of anxiety compared to police officers without depression. Furthermore, after controlling for potential confounders, anxiety was a significant predictor of depression among military police officers.

The prevalence of depression in military police officers in the present study is considerably higher when compared to the general population^(9)^, and is similar to that found in other specific groups, such as university students^(10)^. According to Global Burden of Disease, a resource that compiles comprehensive data and analysis on worldwide trends in global health, the worldwide prevalence of depression in the general population was 4.36% in 202^(11)^. In Brazil, the prevalence of depression in the general population was assessed at 10.27% in 2019, according to data from the Brazilian National Health Survey^(12)^, This value is considerably lower than that observed in the present study (34.5%).

In relation to police officers, the prevalence of depression in the present study was also substantially higher when compared to other studies. A systematic review and meta-analysis study identified a pooled prevalence of 14.6% for depression in police officers^(13)^. Research carried out in the United Kingdom, with a representative sample of the police force, reported a prevalence of depression of 9.8%, making it the most prevalent mental disorder in the force^(14)^.

The differences observed between studies may be related to the nature of professional practice and violence rates in different countries. Brazil, whose population is equivalent to 2.7% of the global population, accounts for one fifth of the world’s homicide rates, which may significantly contribute to the increased prevalence of depression among military police officers, due to high stress and risks associated with the work environment^(15)^.

Depression in police officers is a multifaceted phenomenon, resulting from several factors. Constant exposure to trauma, such as dealing with violent situations and encountering the bodies of people who have recently died, contributes significantly to the development of mental health problems. Studies indicate that suffering related to post-traumatic stress disorder, anxiety and depression tends to increase over time, especially in cases of continuous exposure in the workplace and comorbid health condition^(16)^.

Regarding anxiety, female police officers in this study had significantly higher averages than male officers. This finding is consistent with previous research^(1,17)^, who reported facing unique stressors in police departments that may be associated with higher levels of workload and burnout. Additionally, these studies found that lack of social support and lack of combat experience are associated with officers’ mental health. In addition to the stress inherent in police work, female officers face many challenges, including higher levels of harassment, prejudice, underestimation of physical abilities, discrimination, and a hostile work environment. These factors, coupled with childcare and household management, predispose officers to mental health disorders.

In this study, police officers aged 40 or older had significantly higher anxiety scores compared to younger officers. There was a difference in anxiety scores in relation to the frequency of physical activity, with those who did not practice physical activity having higher anxiety scores when compared to those who did. One possible explanation for these results may be related to the negative effect of aging on the body composition of police officers, which has been previously demonstrated^(18)^. It is important to remember that police officers may be required to initiate a chase, jump, overcome the resistance of a belligerent, make quick and high-risk decisions, among other tasks. To perform these tasks, police officers need to be in good physical condition, fit, and in good general health. The body composition of police officers influences their physical fitness and can allow them to perform better and be healthier^(19)^.

However, the protective effect of physical activity is known. In population samples of healthy individuals, higher levels of objectively measured physical activity are associated with lower levels of depressive and anxiety symptoms^(20)^.

Of the participants in this study, 60.1% reported using alcohol and 11.7% used tobacco. These percentages are higher than those found in a survey of 497 Tanzanian police officers that assessed the prevalence of substance use and potential related disorders among police officers. Approximately 40.2% and 31.4% of participants reported having used one or more substances at some point in their lives and recently, respectively. The most commonly used substance recently was alcohol (31.3%), followed by tobacco (6.2%)^(21)^.

Among police officers, substance use as a means of alleviating current or past distressing experiences is a harmful form of avoidance coping. Previous research, such as that conducted by Arble, Daugherty, and Arnetz^(22)^ and Ballenger *et al*.^(23)^, have reported problematic alcohol use among police officers, possibly related to job stressors and a specific police subculture. These coping strategies become even more complex when they interact with a variety of demographic factors^(22)^. In our study, we observed an association between depression and variables such as sex, physical activity, presence of chronic diseases and medication daily use.

The results of this study demonstrated that police officers with depression presented significantly higher levels of anxiety when compared to police officers without depression. In addition, an important result of the study indicates anxiety as a significant predictor of depression among military police officers.

The relationship between anxiety and depression is complex and raises ongoing debate about the causal relationships and comorbidities between these conditions. A systematic review and meta-analysis examined the longitudinal relationship between anxiety and depression, and the results demonstrated that anxiety symptoms predict subsequent depressive disorders and vice versa. Although a bidirectional relationship was identified for most studies, anxiety symptoms most strongly predicted depressive symptoms. However, the difference in effect size for this analysis was small and possibly not clinically significant^(24)^.

Some conceptual models suggest that anxiety often precedes depression, viewing depression as a dynamic process that begins with an anxious state and evolves into a depressive disorder^(25)^. Furthermore, the comorbidity between anxiety and depression has been widely documented in the literature, and may be justified by the fact that they share some nonspecific components in common, such as negative affectivity^(26)^.

It is important to highlight that this study is one of the first to analyze the relationship between anxiety and depression levels among military police officers, standing out for its representative sample of this population in the Brazilian context. The research adopted multivariate statistical analyses to control confounding variables and accurately assess the impact of anxiety levels on depression. This robust methodological approach strengthens the validity of the results obtained, contributing to the understanding of this complex relationship in a specific occupational group.

### Study limitations

The results of this study need to be interpreted with some limitations in mind. Using self-administered questionnaires to collect data, especially in online surveys, can be challenging due to social desirability bias, which compromises the accuracy of estimates of the prevalence of sensitive behaviors. While this approach was chosen to ensure confidentiality and encourage transparent responses from participants, it also faces the risk of underreporting, particularly in situations that violate the police code of conduct.

### Contributions to nursing, health or public policy

Based on the results of this research, it is possible to think of practical applications that can significantly contribute to mitigating the identified risk factors, promoting a healthier, more productive and safer work environment for military police officers.

First, it is essential to encourage regular physical activity among police officers, highlighting the proven benefits in reducing levels of anxiety and depression. Implementing physical exercise programs adapted to police work’s specific demands aims to improve body composition, physical fitness and general health of police officers. Furthermore, mindfulness is another low-density practice that can be offered to the force, as it is effective in reducing levels of anxiety and depression, as shown in a recent study^(27)^. Promoting an active lifestyle can contribute to these professionals’ psychological and physical well-being.

Subsequently, it is important to strengthen social support networks within police institutions. Creating a positive and supportive work environment can help mitigate the negative effects of stress. It is important to develop internal policies that address and reduce specific stressors faced by police officers, such as harassment, discrimination, and hostile work environments. A healthy and inclusive work environment is essential for police officers’ mental health. In this sense, evidence has already shown that, in general populations of police officers, factors correlated with depression and anxiety include organizational support and recognition of the challenging nature of the work, as well as the existence of a support network outside the work environment^(16)^.

Providing continuing training on stress management and coping strategies can equip officers with the tools they need to deal with the demands of their job. Providing psychological and physical support is also vital to improving officers’ well-being and performance. Continuing training can foster a culture of care and prevention within institutions.

Finally, implementing regular assessments of officers’ mental and physical health to identify symptoms of anxiety and depression early is critical. Mental health interventions should be ongoing and accessible over the long term, allowing officers to access supports as needed, since the impact of trauma can manifest or intensify long after the initial event^(16)^. Continuous monitoring of officer health enables more effective and personalized interventions, promoting a healthier and more resilient police force.

## CONCLUSIONS

The results demonstrate that anxiety and depression are significant problems among police officers in the state of Goiás, Brazil. Anxiety was more pronounced among women, older officers, those who did not exercise regularly, smokers, and those with chronic illnesses and medication daily use. Furthermore, depression was associated with higher levels of anxiety, and increased levels of anxiety were shown to be a significant predictor of depression.

Based on the findings of this study, it is imperative to conduct further research to detail the patterns and identify predictors of stressors faced by military police officers in Brazil. It is essential to unravel cause-and-effect relationships that cannot be detected in cross-sectional studies. It is recommended that prospective studies be conducted with military police recruits from the beginning of their training, followed by longitudinal designs that allow for repeated assessments throughout their career. This ongoing monitoring should include investigations into behavioral changes, psychological history, presence of chronic diseases, lifestyle habits, and patterns of alcohol and other substance use. In addition, it is essential to establish preventive care programs that promote healthy living even before candidates enter the military police, thus ensuring a more prepared and resilient police force.

## Data Availability

The research data are available within the article.
