# Supplementary material for: Prevalence of depression and analysis of its association with anxiety in military police officers: a cross-sectional study
Source: Rev Bras Enferm. 2025 Dec 8;78(5):e20240312. doi: 10.1590/0034-7167-2024-0312 (PMC12707776; doi:10.1590/0034-7167-2024-0312)
Supplement: Translation

# Translation: Prevalência de depressão e análise da relação com a ansiedade em policiais militares: estudo transversal

**DOI:** 10.1590/0034-7167-2024-0312pt

**Authors:** Renato Canevari Dutra da Silva, Carolina Veras Mendes, Maria Neyrian de Fátima Fernandes, Gilson Gonçalves Silva, Lidiane Bernardes Faria Vilela, Elton Brás Camargo

**Affiliations:** I Universidade de Rio Verde. Rio Verde, Goiás, Brasil; II Universidade Federal do Maranhão. Imperatriz, Maranhão, Brasil

**Keywords:** Polícia, Saúde Mental, Depressão, Ansiedade, Transtornos Mentais.

## Abstract

**Objetivos:**

identificar a prevalência de depressão e sua associação com os níveis de ansiedade entre policiais militares.

**Métodos:**

estudo transversal, realizado com policiais militares ativos, utilizando o Inventário de Depressão de Beck e o Inventário de Ansiedade de Beck, ambos validados para o contexto brasileiro. Análises de regressão logística multivariada verificaram associações.

**Resultados:**

amostra foi composta por 672 policiais militares, com prevalência de depressão de 34,5%. Policiais com depressão apresentaram níveis de ansiedade significativamente mais altos. A ansiedade foi um preditor significativo da depressão, com cada aumento de um ponto no escore de ansiedade se associando a um aumento de 1,20 vezes nas chances de depressão (*Odds Ratio* ajustado = 1,20; Intervalo de Confiança de 95% 1,16-1,24; p < 0,001).

**Conclusões:**

a elevada prevalência de depressão e sua associação com a ansiedade destacam a necessidade de implementar ações de prevenção e promoção da saúde mental entre policiais militares.

## INTRODUÇÃO

A profissão de policial militar pode impor uma significativa carga psicológica. Cada chamada policial é imbuída da perspectiva de se deparar com situações emocionalmente desafiadoras, exemplificadas por frequentes chamadas de serviço traumáticas, abrangendo ocorrências como casos de abuso infantil, violência doméstica, acidentes automobilísticos e homicídios^(1)^. Por isso, é imperativo reconhecer que um ambiente ocupacional estressante tem o potencial de precipitar diversas complicações de saúde física e psicológica.

Evidências científicas^(1,2)^ têm mostrado que policiais apresentam algum tipo de problema de saúde mental enquanto estão no trabalho, mas mesmo aqueles que relatam estresse frequente e saúde mental precária têm pouca probabilidade de solicitarem afastamento do trabalho por causa do estigma relacionado ao adoecimento mental na corporação. Além disso, os policiais frequentemente apresentam sintomas de depressão e ansiedade^(3)^. A presença de altos níveis de ansiedade e sintomas depressivos pode resultar em consequências graves, como o suicídio. Dados indicam que o suicídio entre policiais é um grave problema de saúde pública, com 85 casos registrados em 2022 entre policiais militares da ativa^(4)^.

A sobrecarga mental também exerce influência sobre o organismo, podendo manifestar-se por meio de sintomas físicos, tais como um aumento nas lesões ocupacionais e no número de diagnósticos relacionados ao estresse, como hipertensão arterial ou úlceras gastrointestinais. Os estressores policiais e suas implicações mentais e físicas prejudicam a organização policial a longo prazo, resultando na redução do desempenho, aumento do absenteísmo e intensificação da agressividade dos policiais^(5)^.^.^

Ainda há escassez de pesquisas sobre os problemas psicológicos de policiais militares no Brasil, e há a necessidade da realização de pesquisas que avaliem os fatores que influenciam a saúde mental dos policiais^(6)^. Portanto, conduzir pesquisas para aprender mais sobre o perfil de saúde mental dessa população é fundamental. Estudo dessa natureza contribui para o corpo limitado de literatura existente que investiga a prevalência de depressão e ansiedade em policiais militares.

## OBJETIVOS

Identificar a prevalência de depressão e a associação com ansiedade entre policiais militares.

## MÉTODOS

### Aspectos éticos

A presente pesquisa foi aprovada pelo Comitê de Ética em Pesquisa da instituição proponente, e seguiu os preceitos éticos estabelecidos pelas Resoluções n^o^ 466/2012 e n^o^ 588/2018. Os participantes da pesquisa disponibilizaram o consentimento por meio da assinatura do Termo de Consentimento Livre e Esclarecido.

### Desenho, período e local de estudo

Trata-se de estudo de corte transversal, inferencial e quantitativo, realizado com policiais militares ativos atuantes no estado de Goiás. O estado de Goiás, localizado na região Centro-Oeste do Brasil, possui uma população de 7.056,495 habitantes, sendo o 11º estado mais populoso do país e possuindo um contingente de 10.987 policiais militares. O período da coleta de dados ocorreu de dezembro de 2021 a janeiro de 2022.

A amostra de conveniência foi recrutada a partir dos 18 Comandos Regionais de Polícia Militar (CRPM) de Goiás, os quais abrangem todas as microrregiões do estado. A pesquisa foi realizada de forma *online* com bases nos protocolos *STrengthening the Reporting of OBservational studies in Epidemiology* e *CHEcklist for Reporting Results of Internet E-Surveys*.

### População ou amostra; critérios de inclusão e exclusão

Os critérios de elegibilidade para o estudo foram ser profissionais da Polícia Militar de Goiás que estavam na ativa e ocupavam funções em um dos 18 CRPM. Como critério de exclusão, foram definidos os policiais que relataram estar em tratamento atual para algum transtorno mental. Por se tratar de uma amostra de conveniência, avaliou-se o poder amostral após a coleta dos dados. Para o cálculo do poder amostral, foram levadas em consideração as análises de estatísticas de regressões logísticas, assumindo que a ansiedade tem influência moderada na depressão (*Odds Ratio* = 1,2) e proporção de depressão em torno de 20%. A amostra obtida no presente estudo foi de 672 policiais em uma população de 10.987 policiais no estado de Goiás, e ao considerar essas variáveis, o resultado do poder amostral foi de 97%, o que significa que o estudo tem alta probabilidade de detectar um efeito significativo, caso ele exista com um nível de confiança de 95%.

### Protocolo do estudo

A coleta de dados foi realizada por meio de plataforma digital. O convite para participar da pesquisa foi enviado a todos os comandantes responsáveis pelos 18 CRPM de Goiás. Os comandantes, por sua vez, foram encarregados de encaminhar as informações referentes aos trâmites da pesquisa e o endereço eletrônico para preenchimento dos formulários aos policiais sob sua jurisdição.

Os instrumentos de coleta de dados foram estruturados em uma plataforma digital (*Google Forms* ^®^) com as avaliações organizadas em três blocos: características sociodemográficas, laborais, clínicas e estilo de vida; Inventário de Depressão de Beck II; Inventário de Ansiedade de Beck.

Em relação às características sociodemográficas, laborais, clínicas e estilo de vida, o instrumento foi elaborado pelos autores da pesquisa com questões sociodemográficas sobre a idade, sexo e situação conjugal. As variáveis laborais foram avaliadas pela identificação da patente, função exercida na corporação, tempo de serviço e renda. O estilo de vida dos policiais foi avaliado pela prática de atividade física (atividade praticada por no mínimo 30 minutos), uso de álcool e tabaco. Em relação aos aspectos clínicos, foram avaliados a presença de doença crônica e o uso de medicação diária.

Inventário de Depressão de Beck II (em inglês, *Beck Depression Inventory II* (BDI-II)): a depressão foi avaliada pelo BDI-II, instrumento validado para o contexto brasileiro e amplamente utilizado para avaliar a gravidade da depressão em amostras clínicas e não clínicas^(7)^. O instrumento é composto por 21 questões que avaliam sintomas como tristeza, culpa, desesperança, alterações no sono e apetite, entre outros, refletindo os componentes somáticos, cognitivos e afetivos da depressão. A pontuação do instrumento varia de 0 a 63, a depender da intensidade do sintoma vivenciado na última semana, nos quais pontuações mais altas indicam maior gravidade da depressão. Conforme indicado por Gomes-Oliveira *et al.* ^(7)^, os policiais militares foram categorizados em dois grupos com base no BDI-II: ausência de depressão, com pontuações iguais ou inferiores a 10, e presença de depressão, com pontuações iguais ou superiores a 11. Os resultados de confiabilidade do instrumento na presente amostra demonstraram um coeficiente alfa de Cronbach de 0,89 e um coeficiente ômega de McDonald de 0,89.

Inventário de Ansiedade de Beck (em inglês, *Beck Anxiety Inventory* (BAI)): a ansiedade foi avaliada pelo BAI, que é um instrumento que consiste em 21 itens que descrevem os sintomas da ansiedade, como medo, tremores, sudorese, nervosismo, sensações de pânico, entre outros. O BAI foi validado para o Brasil, e cada item do instrumento é pontuado de 0 a 3, no qual 0 indica ausência do sintoma e 3 indica grave sintomatologia^(8)^. A pontuação varia de 0 a 63, com pontuações mais altas refletindo maiores níveis de ansiedade. Devido à natureza latente da ansiedade e à sua variação em diferentes níveis, ela foi avaliada como uma variável contínua. Os testes de confiabilidade do BAI, no presente estudo, apresentaram coeficientes de alfa de Cronbach de 0,92 e de ômega de McDonald de 0,93.

### Análises dos resultados e estatística

O banco de dados foi construído por meio de estratégia de dupla digitação no *software Excel* ^®^ e, posteriormente, exportado para o *software R Studio*, versão 2024.04.1. As análises realizadas incluíram estatística descritivas por meio da apresentação de frequência absoluta (n) e relativa (%), além da média (M) e desvio padrão (±DP) das variáveis contínuas. Para avaliar as diferenças entre as médias dos níveis de ansiedade, foram realizados testes de Mann-Whitney e Kruskal-Wallis, em virtude de não apresentarem distribuição normal. Para as análises de associações entre a depressão e as variáveis independentes, foi realizado o teste qui-quadrado. Foram consideradas diferenças e associações significativas o nível de significância de 5% (p < 0,05).

Com o objetivo de discernir o impacto singular da ansiedade sobre a depressão, procedeu-se a uma análise multivariada, controlando as variáveis associadas à depressão nas análises bivariadas. A magnitude da relação entre ansiedade e depressão foi avaliada por meio de regressão logística multivariada, utilizando o *Odds Ratio* ajustado (ORa) e um Intervalo de Confiança de 95% (IC95%), em que valores de p menores que 0,05 foram considerados estatisticamente significativos. Os pressupostos da regressão logística multivariada foram avaliados por meio das métricas de fator de inflação da variância (VIF) e *tolerance*. Os resultados da VIF variaram entre 1,01 e 1,51, e os de *tolerance*, entre 0,65 e 0,98, acatando, portanto, os pressupostos da regressão logística multivariada.

## RESULTADOS

A amostra da presente pesquisa foi composta por 672 policiais militares da ativa do estado de Goiás, Brasil. A maior parte dos participantes é masculina (90,62%), com idade predominante entre 30 e 39 anos (47,32%), casada (77,53%) e atuando principalmente em funções operacionais (76,78%), com mais de seis anos de serviço (76%). A renda majoritária situa-se entre R$5.000,00 e R$10.000,00 (65,32%). Em relação à atividade física, 21,57% relataram não praticar nenhum nível de atividade. O uso atual de tabaco foi relatado por 11,75%, e de álcool, por 88,24% da amostra avaliada. A maioria não possui doenças crônicas (68,45%) ou necessidade de medicação diária (71,87%). Em relação à depressão, 232 (34,5%; IC95% 31 - 37,9) foram categorizados com depressão, e 440 (65,5%; IC95% 62,1 - 69) não apresentaram sintomas significativos de depressão. Os escores de depressão e ansiedade apresentaram médias de 9,47 (±7,94) e 10,37 (±9,55), respectivamente (Tabela 1).

**Tabela 1:** Características sociodemográficas, laborais, clínicas e estilo de vida de policiais militares, Goiás, Brasil, 2021-2022

Variáveis	n	%
Sexo		
Feminino	63	9,37
Masculino	609	90,62
Idade		
Até 29 anos	65	9,67
30 a 39 anos	318	47,32
40 anos ou mais	289	43
Situação conjugal		
Casado	521	77,53
Solteiro, divorciado ou viúvo	151	22,47
Patente policial		
Praças (soldado a aspirante)	561	83,5
Oficiais (tenente a coronel)	111	16,5
Função policial atual		
Administrativa	156	23,21
Operacional	516	76,78
Tempo de serviço policial		
Até 5 anos	161	24
6 anos ou mais	511	76
Renda		
Até R$5.000,00	112	16,66
R$5.000,00 a R$10.000,00	439	65,32
R$10.000,00 a R$15.000,00	66	9,82
R$15.000,00 ou mais	55	8,18
Atividade física		
Três vezes ou mais por semana	283	42,11
Até duas vezes na semana	244	36,31
Não pratica	145	21,57
Uso de tabaco		
Não	593	88,24
Sim	79	11,75
Uso de bebida alcóolica		
Não	268	39,88
Sim	404	60,11
Doença crônica		
Não possui	460	68,45
Hipertensão arterial	61	9,07
Diabetes mellitus	11	1,63
Outros	140	20,83
Uso de medicação diária		
Não	483	71,87
Sim	189	28,12
Depressão		
Ausente	440	65,5
Presente	232	34,5
-	**Média**	**Desvio padrão**
Escore da BDI	9,47	7,94
Escore da BAI	10,37	9,55

*BDI - Inventário de Depressão de Beck; BAI - Inventário de Ansiedade de Beck.*

As análises das diferenças de médias dos escores de ansiedade, avaliado pelo BAI, e das associações de depressão, avaliadas pelo BDI, são apresentadas na Tabela 2. Em relação à ansiedade, as mulheres apresentaram média significativamente maior (13,44; ±9,11) do que os homens (10,04; ±9,55) (p < 0,001). Os policiais militares com 40 anos ou mais apresentaram escores significativamente maiores quando comparados aos mais jovens (p = 0,04). Houve diferença das médias de ansiedade em relação à frequência da prática de atividade física (p < 0,001), sendo que aqueles que não praticam atividade física apresentaram maiores escores (14,40; ±20,93), quando comparados aos praticantes. Os policiais que relataram fazer uso de tabaco apresentaram média significativamente maior (22,67; ±9,02), em comparação aos não fumantes (10,19; ±9,61) (p = 0,04). A presença de doença crônica e uso de medicação diária foi associada a níveis significativamente mais altos de ansiedade (p < 0,001). Em relação à depressão, observou-se associação com sexo, prática de atividade física, doença crônica e uso de medicação diária (p < 0,05) (Tabela 2).

**Tabela 2:** Diferenças de médias dos escores de ansiedade e associações com depressão de acordo com as características sociodemográficas, laborais, clínicas e estilo de vida de policiais militares, Goiás, Brasil, 2021-2022

Variáveis	Ansiedade		Depressão		Valor de *p* ^**^
	Média ()	Valor de *p* ^*^	Ausente n (%)	Presente n (%)	
Sexo		<0,001			0,04
Feminino	13,44 (9,11)		34 (7,7)	29 (12,50)	
Masculino	10,04 (9,55)		406 (92,27)	203 (87,05)	
Idade		0,04			0,58
Até 29 anos	8,84 (7,02)		46 (10,45)	19 (8,19)	
30 a 39 anos	9,76 (9,09)		204 (46,36)	114 (49,13)	
40 anos ou mais	11,37 (10,42)		190 (43,18)	99 (42,67)	
Situação conjugal		0,52			0,57
Casado	10,33 (9,26)		344 (78,18)	177 (76,29)	
Solteiro, divorciado ou viúvo	10,16 (10,52)		96 (21,81)	55 (23,70)	
Patente policial		0,09			0,06
Praças (soldado a aspirante)	10,57 (9,59)		359 (81,59)	202 (87,06)	
Oficiais (tenente a coronel)	9,29 (9,30)		81 (18,40)	30 (12,93)	
Função policial atual		0,30			0,54
Administrativa	10,92 (9,37)		99 (22,50)	57 (24,56)	
Operacional	10,19 (9,61)		341 (77,50)	175 (75,43)	
Tempo de serviço policial		0,76			0,93
Até 5 anos	9,60 (8,18)		105 (23,86)	56 (24,13)	
6 anos ou mais	10,60 (9,94)		335 (76,13)	176 (75,86)	
Renda		0,07			0,13
Até R$5.000,00	11,83 (10,49)		65 (14,77)	47 (20,25)	
R$5.000,00 a R$10.000,00	10,30 (9,17)		287 (65,22)	152 (65,51)	
R$10.000,00 a R$15.000,00	9,72 (10,31)		48 (10,90)	18 (7,75)	
R$15.000,00 ou mais	8,63 (9,43)		40 (9,09)	15 (6,46)	
Atividade física		<0,001			<0,001
Três vezes ou mais por semana	8,43 (8,23)		203 (46,13)	80 (34,48)	
Até duas vezes na semana	10,20 (9,40)		163 (37,04)	81 (34,91)	
Não pratica	14,40 (10,93)		74 (16,81)	71 (30,60)	
Uso de tabaco		0,04			0,14
Não	10,19 (9,61)		394 (89,54)	199 (85,77)	
Sim	22,67 (9,02)		46 (10,55)	33 (14,22)	
Uso de bebida alcóolica		0,47			0,05
Não	10,13 (9,52)		187 (42,50)	81 (34,91)	
Sim	10,51 (9,58)		253 (57,50)	151 (65,08)	
Doença crônica		<0,001			<0,001
Não possui	8,36 (8,14)		340 (77,27)	120 (51,72)	
Hipertensão arterial	12,59 (10,37)		38 (8,63)	23 (9,91)	
Diabetes mellitus	15,72 (12,24)		6 (1,63)	5 (2,15)	
Outros	15,56 (10,93)		56 (12,72)	84 (36,20)	
Uso de medicação diária		<0,001			<0,001
Não	8,63 (8,39)		347 (78,86)	136 (58,62)	
Sim	14,79 (10,85)		93 (21,13)	96 (41,37)	

* Valor de p para o teste U de Mann-Whitney ou Kruskal-Wallis;

** Valor de p para o teste qui-quadrado.

A Figura 1 ilustra a diferença entre as médias dos escores de ansiedade entre os policiais militares de acordo com a ausência ou presença de depressão. Os policiais com depressão apresentaram média de ansiedade significativamente maior (18,33; ±10,28), quando comparados aos policiais sem depressão (6,16; ±5,71) (p < 0,001).

**Figura 1:** Relação entre depressão e os escores de ansiedade em policiais militares, Goiás, Brasil, 2021-2022

*Nota: círculos indicam pontuação média, e barras superiores e inferiores indicam Intervalos de Confiança de 95% para cada grupo de policiais.*

As variáveis independentes significativamente associadas à depressão foram inseridas em um modelo multivariado com o intuito de investigar suas relações. A presença de outras doenças crônicas mostrou-se significativa como preditor da depressão. Os resultados da regressão logística multivariada demonstraram que, mesmo após a inserção de possíveis fatores de confusão, a ansiedade continua a ser um preditor significativo da depressão. Cada aumento de uma unidade no escore de ansiedade está associado ao aumento de 1,20 vezes nas chances de depressão entre os policiais militares (ORa = 1,20; IC95% 1,16 - 1,24; p < 0,001) (Tabela 3).

**Tabela 3:** Análise de regressão logística multivariada da associação entre depressão e ansiedade controlando as características sociodemográficas, clínicas e estilo de vida, Goiás, Brasil, 2022

Variáveis	ORa (IC95%)	Valor de *p*
Sexo		
Feminino	1,12 (0,58 - 2,17)	0,72
Atividade física		
Até duas vezes na semana	0,89 (0,55 - 1,42)	0,63
Não pratica	1,11 (0,64 - 1,92)	0,70
Doença crônica		
Hipertensão arterial	0,81 (0,35 - 1,88)	0,63
Diabetes mellitus	0,88 (0,16 - 4,65)	0,88
Outros	2,13 (1,23 - 3,68)	0,007
Uso de medicação diária		
Sim	1,05 (0,59 - 1,85)	0,86
Ansiedade		
Escore da BAI	1,20 (1,16 - 1,24)	<0,001

*ORa - Odds Ratio ajustado; IC95% - Intervalo de Confiança de 95%.*

## DISCUSSÃO

Os resultados do estudo demonstraram prevalência significativa de depressão entre os policiais militares. Os policiais com depressão apresentaram níveis de ansiedade significativamente maiores, em comparação aos policiais sem depressão. Além disso, após o controle de possíveis fatores de confusão, a ansiedade foi um preditor significativo da depressão entre os policiais militares.

A prevalência de depressão em policiais militares no presente estudo é consideravelmente maior quando comparada à população geral^(9)^, e é semelhante ao encontrado em outros grupos específicos, como estudantes universitários^(10)^. De acordo com *Global Burden of Disease*, um recurso que compila dados e análises abrangentes sobre as tendências mundiais na saúde global, a prevalência mundial de depressão na população geral foi de 4,36% em 2021^(11)^. No Brasil, a prevalência de depressão na população geral foi avaliada em 10,27% em 2019, de acordo com dados da Pesquisa Nacional de Saúde^(12)^, valor este consideravelmente inferior ao observado no presente estudo (34,5%).

Em relação aos policiais, a prevalência de depressão no presente estudo também foi substancialmente maior, quando comparado a outras pesquisas. Estudo de revisão sistemática e meta-análise identificou prevalência agrupada de 14,6% para a depressão em policiais^(13)^. Pesquisa realizada no Reino Unido, com amostra representativa da força policial, relatou prevalência de depressão de 9,8%, sendo o transtorno mental mais prevalente na corporação^(14)^.

As diferenças observadas entre os estudos podem estar relacionadas à natureza da prática profissional e pelos índices de violência nos diferentes países. No Brasil, cuja população equivale a 2,7% da população global, ocorre um quinto das taxas mundiais de homicídios, o que pode contribuir significativamente para o aumento da prevalência de depressão entre policiais militares, devido ao estresse elevado e aos riscos associados ao ambiente de trabalho^(15)^.

A depressão em policiais é um fenômeno multifacetado, resultante de diversos fatores. A exposição constante a traumas, como a gestão de situações violentas e o encontro com corpos de pessoas que tenham falecido recentemente, contribui de maneira significativa para o desenvolvimento de problemas de saúde mental. Estudos indicam que o sofrimento relacionado ao transtorno de estresse pós-traumático, ansiedade e depressão tende a aumentar com o tempo, especialmente em casos de exposição contínua no ambiente de trabalho e condições de saúde comórbidas^(16)^.

No que se refere à ansiedade, as policiais do sexo feminino deste estudo apresentaram médias significativamente maiores do que os homens. Esta constatação é consistente com pesquisas anteriores^(1,17)^, que relataram o enfrentamento de estressores únicos nos departamentos de polícia, os quais podem estar associados a níveis mais altos de sobrecarga de trabalho e esgotamento. Além disso, essas pesquisas descobriram que a falta de suporte social e a falta de experiência em combate estão associadas à saúde mental dos policiais. Além do estresse inerente ao trabalho policial, as policiais enfrentam muitos desafios, como níveis mais altos de assédio, preconceito, subestimação das habilidades físicas, discriminação e um ambiente de trabalho hostil. Esses fatores, somados aos cuidados com os filhos e à administração do lar, predispõem os policiais aos transtornos mentais.

Neste estudo, os policiais com 40 anos ou mais apresentaram escores de ansiedade significativamente maiores, em comparação aos mais jovens. Houve uma diferença nas médias de ansiedade em relação à frequência da prática de atividade física, sendo que aqueles que não praticam atividade física apresentaram maiores escores de ansiedade, quando comparados aos praticantes. Uma possível explicação para esses resultados pode estar relacionada ao efeito negativo do envelhecimento na composição corporal dos policiais, que já foi evidenciado anteriormente^(18)^. É importante lembrar que os policiais podem ser requisitados a iniciar uma perseguição, pular, superar a resistência de um beligerante, tomar decisões rápidas e de alto risco, entre outras. Para realizar essas tarefas, os policiais precisam de um bom condicionamento físico, aptidão física e saúde geral. A composição corporal dos policiais influencia a aptidão física e pode permitir que eles tenham um desempenho melhor e sejam mais saudáveis^(19)^.

Todavia, sabe-se do efeito protetor que a atividade física proporciona. Em amostras populacionais de indivíduos saudáveis, níveis mais elevados de atividade física mensurada objetivamente estão associados a níveis mais baixos de sintomas depressivos e ansiosos^(20)^.

Dos participantes deste estudo, 60,1% relataram consumir álcool e 11,7% faziam uso de tabaco. Esses porcentuais são maiores do que os encontrados em pesquisa realizada com 497 policiais da Tanzânia que avaliou a prevalência do uso de substâncias e potenciais transtornos relacionados entre os policiais. Aproximadamente 40,2% e 31,4% dos participantes relataram ter usado uma ou mais substâncias alguma vez na vida e recentemente, respectivamente. A substância mais comumente usada recentemente foi o álcool (31,3%), seguido pelo tabaco (6,2%)^(21)^.

Entre os policiais, o uso de substâncias como meio de atenuar experiências angustiantes presentes ou passadas é uma forma prejudicial de enfrentamento por evitação. Investigações anteriores, como as realizadas por Arble, Daugherty e Arnetz^(22)^ e Ballenger *et al*.^(23)^, têm relatado o uso problemático de álcool entre policiais, possivelmente relacionado aos estressores do trabalho e a uma subcultura específica da polícia. Essas estratégias de enfrentamento se tornam ainda mais complexas quando interagem com diversos fatores demográficos^(22)^. Em nosso estudo, observamos uma associação entre a depressão e variáveis como sexo, prática de atividade física, presença de doenças crônicas e uso diário de medicação.

Os resultados do presente estudo demonstraram que os policias com depressão apresentaram níveis de ansiedade significativamente mais elevados quando comparados aos policiais sem depressão. Além disso, um resultado importante do trabalho indica a ansiedade como um preditor significativo da depressão entre os policiais militares.

A relação entre ansiedade e depressão é complexa e suscita debates contínuos sobre as relações causais e comorbidades entre essas condições. Estudo de revisão sistemática e meta-análise examinou a relação longitudinal entre ansiedade e depressão, e os resultados demonstraram que os sintomas de ansiedade preveem transtornos depressivos subsequentes e vice-versa. Embora a relação bidirecional foi identificada para a maioria dos estudos, os sintomas de ansiedade previram mais fortemente os sintomas depressivos. No entanto, a diferença no tamanho do efeito para esta análise foi pequena e possivelmente não clinicamente significativa^(24)^.

Alguns modelos conceituais sugerem que a ansiedade frequentemente precede a depressão, considerando a depressão como um processo dinâmico que começa com um estado ansioso e evolui para um transtorno depressivo^(25)^. Além disso, a comorbidade entre ansiedade e depressão tem sido amplamente documentada pela literatura, podendo ser justificada pelo fato de compartilharem alguns componentes inespecíficos em comum, como a afetividade negativa^(26)^.

É importante evidenciar que este estudo é um dos primeiros a analisar a relação entre os níveis de ansiedade e depressão entre policiais militares, destacando-se pela amostra representativa dessa população no contexto brasileiro. A pesquisa adotou análises estatísticas multivariadas para controlar variáveis confundidoras e avaliar, de forma precisa, o impacto dos níveis de ansiedade na depressão. Essa abordagem metodológica robusta fortalece a validade dos resultados obtidos, contribuindo para o entendimento dessa complexa relação em um grupo ocupacional específico.

### Limitações do estudo

Os resultados deste estudo precisam ser interpretados considerando algumas limitações. O uso de questionários autoadministrados para coletar dados, especialmente em pesquisas *online*, pode ser desafiador, devido ao viés de desejabilidade social, que compromete a precisão das estimativas de prevalência de comportamentos sensíveis. Embora esta abordagem tenha sido escolhida para garantir a confidencialidade e incentivar respostas transparentes dos participantes, ela também enfrenta o risco de subnotificação, particularmente em situações que violem o código de conduta policial.

### Contribuições para as áreas da enfermagem, saúde ou políticas públicas

A partir dos resultados da presente pesquisa, é possível pensar em aplicações práticas que podem contribuir significativamente para a mitigação dos fatores de risco identificados, promovendo um ambiente de trabalho mais saudável, produtivo e seguro para os policiais militares.

Em primeiro lugar, é essencial incentivar a prática regular de atividade física entre os policiais, destacando os benefícios comprovados na redução dos níveis de ansiedade e depressão. Implementar programas de exercícios físicos adaptados às demandas específicas do trabalho policial visa melhorar a composição corporal, aptidão física e saúde geral dos policiais. Além disso, o *mindfulness* é outra prática de baixa densidade que pode ser ofertada para a corporação, por ser eficaz na diminuição dos níveis de ansiedade e depressão, conforme apresentado em estudo recente^(27)^. A promoção de estilo de vida ativo pode contribuir para o bem-estar psicológico e físico desses profissionais.

Depois, é importante fortalecer as redes de suporte social dentro das instituições policiais. Criar um ambiente de trabalho positivo e solidário pode ajudar a mitigar os efeitos negativos do estresse. É importante desenvolver políticas internas que abordem e reduzam estressores específicos enfrentados por policiais, como assédio, discriminação e ambientes de trabalho hostis. Um ambiente de trabalho saudável e inclusivo é fundamental para a saúde mental dos policiais. Nesse sentido, as evidências já têm mostrado que, em populações gerais de policiais, os fatores correlacionados com depressão e ansiedade incluem o suporte organizacional e o reconhecimento da natureza desafiadora do trabalho, bem como a existência de uma rede de apoio fora do ambiente de trabalho^(16)^.

Oferecer treinamento contínuo sobre a gestão do estresse e estratégias de enfrentamento pode equipar os policiais com as ferramentas necessárias para lidar com as demandas de sua profissão. Assegurar suporte psicológico e físico também é vital para melhorar o bem-estar e a *performance* dos policiais. A capacitação constante pode promover uma cultura de cuidado e prevenção dentro das instituições.

Por fim, implementar avaliações periódicas da saúde mental e física dos policiais para identificar precocemente sintomas de ansiedade e depressão é fundamental. As intervenções de saúde mental devem ser contínuas e acessíveis a longo prazo, permitindo que os policiais utilizem os suportes conforme necessário, uma vez que o impacto do trauma pode se manifestar ou intensificar muito tempo após o evento inicial^(16)^. O monitoramento contínuo da saúde dos policiais permite intervenções mais eficazes e personalizadas, promovendo uma força policial mais saudável e resiliente.

## CONCLUSÕES

Os resultados demonstram que a ansiedade e a depressão são problemas significativos entre os policiais do estado de Goiás, Brasil. A ansiedade foi mais acentuada entre mulheres, policiais mais velhos, não praticantes de atividade física, fumantes, e aqueles com doenças crônicas e uso diário de medicação. Além disso, a depressão foi associada a níveis mais elevados de ansiedade, e o aumento dos níveis de ansiedade mostrou-se um preditor significativo da depressão.

Com base nos achados deste estudo, é imperativo realizar mais pesquisas para detalhar os padrões e identificar os preditores dos estressores enfrentados pelos policiais militares no Brasil. É essencial desvendar as relações de causa e efeito que não podem ser detectadas em estudos transversais. Recomenda-se a realização de estudos prospectivos com recrutas da polícia militar desde o início de sua formação, acompanhados por desenhos longitudinais que permitam avaliações repetidas durante toda a carreira. Esse acompanhamento contínuo deve incluir investigações sobre mudanças comportamentais, histórico psicológico, presença de doenças crônicas, hábitos de vida e padrões de consumo de álcool e outras substâncias. Além disso, é fundamental estabelecer programas de cuidados preventivos que promovam uma vida saudável antes mesmo do ingresso dos candidatos na polícia militar, garantindo, assim, uma força policial mais preparada e resiliente.

## Data Availability

Os dados da pesquisa estão disponíveis no corpo do artigo.
